# First Complete Genome Sequence of a Genogroup II Genotype 18 Porcine Norovirus, Strain QW125

**DOI:** 10.1128/genomeA.00344-13

**Published:** 2013-06-13

**Authors:** Tomoichiro Oka, Linda J. Saif, Qiuhong Wang

**Affiliations:** Food Animal Health Research Program, Ohio Agricultural Research and Development Center, Department of Veterinary Preventive Medicine, College of Veterinary Medicine, The Ohio State University, Wooster, Ohio, USAa; Department of Virology II, National Institute of Infectious Diseases, Tokyo, Japanb

## Abstract

Noroviruses, members of the family *Caliciviridae*, are genetically diverse. We report the first complete genome sequence of a genogroup II genotype 18 porcine norovirus, strain QW125. A protein BLAST search revealed that identity scores of this strain compared to other norovirus strains were highest in the predicted protease region.

## GENOME ANNOUNCEMENT

Noroviruses (NoVs) have been detected from humans, swine, cattle, sheep, rodents, cats, dogs, and lions (1–4). The NoV genome is a positive-sense, single-stranded RNA molecule with three open reading frames (ORFs). ORF1 encodes 6 to 7 nonstructural proteins (NS1-2, NS3 [NTPase], NS4, NS5 [VPg], NS6 [protease], and NS7 [polymerase]). ORF2 and ORF3 encode major (VP1) and minor (VP2) structural proteins, respectively. Based on the complete VP1 sequences, NoVs are classified into at least five genogroups (GI to GV), which are further divided into multiple genotypes (5). The GII.11, -18, and -19 NoVs were detected from swine (6); however, only two full-length genome sequences of GII.11 (AB126320 and HQ392821), and none for GII.18 and -19, are available in GenBank. We determined the first full-length genome sequence of a porcine GII.18 NoV.

The Po/NoV/GII.18/OH-QW125/2003/US strain (QW125) was detected from the feces of a finisher pig. The 3′-end 3.3-kb cDNA fragment corresponding to the partial polymerase, VP1 and VP2 genes, and 3′-untranslated region (UTR) of QW125 has been determined previously (6). In this study, the 5′-end 4.5-kb fragment corresponding to the 5′-UTR and most of ORF1 was amplified using seminested PCR with forward primer Noro-F1-F (7) and QW125-specific reverse primers designed in the polymerase gene. The 5′-end sequence of the genome was confirmed by 5′ rapid amplification of cDNA ends (5′-RACE) method. The amplified cDNA fragment was cloned into pCR4 Blunt-Topo vector (Invitrogen) and sequenced by primer walking using a set of QW125-specific primers and an automated sequencer, ABI3730 (Applied Biosystems). The full-length genome sequence was assembled using the Sequencher 4.10.1 program (GeneCodes).

The genome of QW125 consists of 7,612 nucleotides (nt), excluding the poly(A) tail. Similar to other NoVs, the genome was predicted to encode three ORFs, nucleotide positions 8 to 5083 (ORF1), 5064 to 6737 (ORF2), and 6737 to 7564 (ORF3). The 5′- and 3′-UTRs were 7 nt and 48 nt long, respectively. The ORF1-encoded polyprotein was predicted to be protease processed to NS1-2 (amino acid [aa] 1 to 327), NS3 (aa 328 to 693), NS4 (aa 694 to 867), NS5 (aa 868 to 1000), NS6 (aa 1001 to 1181), and NS7 (aa 1182 to 1691) based on the cleavage sites, characteristic motifs, and protein sizes (8). The first three aa residues of polyprotein and VP1 were MMA and MMM, respectively, and differed from those (MKM) of other complete genomes available for NoV GII strains. A protein BLAST search revealed that the highest identity scores of each region of the QW125 strain to those of other human and animal NoV strains were 63% (NS1-2), 84% (NS3), 48% (NS4), 84% (NS5), 91% (NS6), 82% (NS7), 71% (VP1), and 57% (VP2), respectively. The putative NS6 (protease) region had the highest aa identity, suggesting a strict evolutionary constraint of this region.

The accumulation of complete genome sequences of multiple genogroups and genotypes of NoVs is useful to establish new classification schemes, assess the potential interspecies transmission of NoVs, and investigate the viral protein functions, virus-host interactions, and pathogenesis of NoVs.

### Nucleotide sequence accession number.

The genome sequence of the QW125 strain has been deposited in GenBank under the accession no. AY823305.
